# Case Report: Two cases of apparent discordance between non-invasive prenatal testing (NIPT) and amniocentesis resulting in feto-placental mosaicism of trisomy 21. Issues in diagnosis, investigation and counselling

**DOI:** 10.3389/fgene.2022.982508

**Published:** 2022-10-25

**Authors:** Agnese Feresin, Tamara Stampalija, Stefania Cappellani, Rossana Bussani, Flavio Faletra, Flora Murru, Sheila Ulivi, Sarah Suergiu, Pasquale Savarese, Antonio Pedicini, Margherita Policicchio, Raffaella Ruggiero, Barbara Bosio, Giovanni Savarese, Carmela Ardisia

**Affiliations:** ^1^ Department of Medicine, Surgery and Health Sciences, University of Trieste, Trieste, Italy; ^2^ Institute for Maternal and Child Health, IRCCS Burlo Garofolo, Trieste, Italy; ^3^ Unit of Pathologic Anatomy and Histology, Asugi, Trieste, Italy; ^4^ Ames, Centro Polidiagnostico Strumentale, Naples, Italy; ^5^ AORN, San Giuseppe Moscati, Avellino, Italy

**Keywords:** NIPT, false negative cffDNA, feto-placental mosaicism, trisomy 21, SNP array, autopsy

## Abstract

The sequencing of cell-free fetal DNA in the maternal plasma through non-invasive prenatal testing (NIPT) is an accurate genetic screening test to detect the most common fetal aneuploidies during pregnancy. The extensive use of NIPT, as a screening method, has highlighted the limits of the technique, including false positive and negative results. Feto-placental mosaicism is a challenging biological issue and is the most frequent cause of false positive and negative results in NIPT screening, and of discrepancy between NIPT and invasive test results. We are reporting on two cases of feto-placental mosaicism of trisomy 21, both with a low-risk NIPT result, identified by ultrasound signs and a subsequent amniocentesis consistent with a trisomy 21. In both cases, after the pregnancy termination, cytogenetic and/or cytogenomic analyses were performed on the placenta and fetal tissues, showing in the first case a mosaicism of trisomy 21 in both the placenta and the fetus, but a mosaicism in the placenta and a complete trisomy 21 in the fetus in the second case. These cases emphasize the need for accurate and complete pre-test NIPT counselling, as well as to identify situations at risk for a possible false negative NIPT result, which may underestimate a potential pathological condition, such as feto-placental mosaicism or fetal trisomy. Post-mortem molecular autopsy may discriminate between placental, fetal and feto-placental mosaicism, and between complete or mosaic fetal chromosomal anomalies. A multidisciplinary approach in counselling, as well as in the interpretation of biological events, is essential for the clarification of complex cases, such as feto-placental mosaicisms.

## Introduction

In humans, the most common aneuploidies are trisomies, which represent about 0.3% of all live births and make up an even higher proportion in products of conception (Hassold and Hunt, 2001). The most common human trisomies involve chromosome 21 (T21) and may consist of either a complete trisomy or a mosaic trisomy. T21 is known as Down’s syndrome (DS) and is associated with a specific phenotype, which typically includes brachycephaly, epicanthus, narrow and up-slanted palpebral fissures, flat nose, micrognathia, single palmar fusion crease and sandal gap, as well as systemic clinical manifestations that vary in severity, usually milder in mosaic T21, but always involving mild to moderate cognitive impairment and the possibility of life-threating comorbidities, caused in particular by cardiac or gastrointestinal malformations (Bull, 2020).

The prenatal presentation of T21 is also extremely heterogenous, ranging from precocious miscarriages, early severe malformations, associations of multi-organ conditions and soft markers to milder forms with isolated soft markers, but where there are no major or specific signs or even altered biochemical markers.

Women in their first 3 months of pregnancy are offered first trimester combined screening (FTCS), based on maternal age, fetal nuchal translucency thickness (NT) and serum markers such as beta human chorionic gonadotropin (β-HCG) and pregnancy-associated plasma protein A (PAPP-A), with a detection rate for T21 of 90–95% and a false positive rate of 2.5–5% (Kagan et al., 2019), as well as a positive predicted value of 3.4% (Norton et al., 2015).

Non-invasive prenatal testing (NIPT) is a screening method for the early identification of the most frequent autosomal aneuploidies (trisomies 21, 18 and 13) in the fetus during pregnancy, with high sensitivity and specificity and very high negative predictive values (NPV) (Bianchi and Wilkins-Haug, 2014). NIPT performance is demonstrably superior to FTCS in high-risk cases and also among the general population, particularly in the detection of T21 (Bianchi and Wilkins-Haug, 2014; Gil et al., 2017).

NIPT on cell free fetal-DNA (cffDNA) circulating in the maternal blood has been increasingly used since 2011, and today it is an integral part of clinical practice in many countries. Different policies concerning the proposal and administration of NIPT are currently applied across the world. In Europe, NIPT is currently offered as a first-tier universal screening method in the Netherlands (van der Meij et al., 2019) and in Belgium (Willems et al., 2014) and, in other countries, as a contingent test for women considered to be high-risk after FTCS. In most countries, however, the ease of use and the extensive adoption of NIPT may contribute to the lack of optimal pre and post-test counselling about the potentially controversial results determined by the current technical limitations in the method and in the way data can be interpreted and managed (Gadsbøll et al., 2020). CffDNA analysed by NIPT originates from apoptosis of placental (cytotrophoblast and syncytiotrophoblast) cells and thus represents the molecular identity of extraembryonic tissue (Lo et al., 1997; Flori et al., 2004).

NIPT does not produce a diagnostic result, due to technical and computational limits and also to biological issues. Among the test accuracy limitations are the occurrence of false positive and false negative results, which may occur in the case of multiple pregnancies, vanishing twins, maternal malignancies or mosaicisms. Rarely, mosaicism may reflect a maternal constitutional mosaicism, most frequently related to sexual chromosome aneuploidies (Zhang et al., 2017), or a somatic mosaicism, in the case of an eventual maternal malignancy (Bianchi et al., 2015). More frequently the mosaicism diagnosed through invasive analysis during pregnancy involves the placenta, the fetus or both (Ledbetter et al., 1992; Smidt-Jensen et al., 1993; Malvestiti et al., 2015).

We are reporting on two cases of feto-placental mosaicism of T21. In both cases, the discrepancy between the NIPT and amniocentesis results was consistent with a mosaicism: a feto-placental mosaicism in the first case and a confined placental mosaicismin in the second case.

## Clinical reports

### Case 1

**FIGURE 1:**
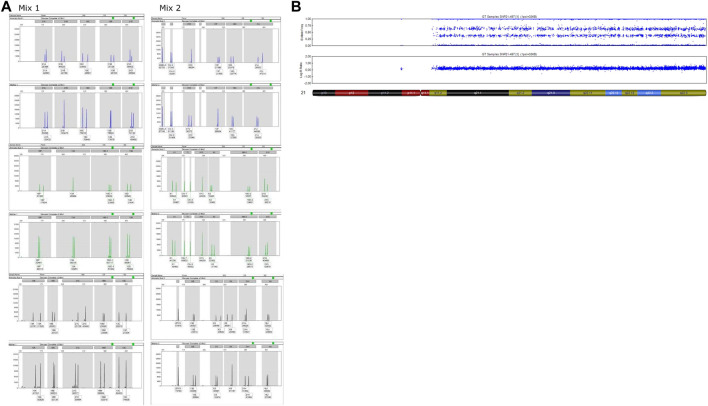
Analyses performed on amniotic fluid of Case 1. **(A)** QF-PCR results. For each channel, the first lane represents the amniotic fluid and the second is the maternal blood sample. Markers 21A, 21B, 21D and 21C show a complete T21. **(B)** SNP-array results. Complete T21.

The case involves the first spontaneous pregnancy of a healthy couple of Caucasian ancestry, with unremarkable personal and family history of both the partners. The woman’s age at conception was 31 and she was a smoker (about 10 cigarettes/day). The first trimester echography performed at the 11th week of gestation (WG) was normal, with a NT measurement of 1.8 mm and nasal bone visualisation. The PAPP-A level was slightly low, at 0.34 MoM, and the FTCS indicated a low risk for the main trisomies and a specific risk of 1:1,326 for trisomy 21. The couple performed NIPT at 12 WG for their own choice in accordance with the referring physician, which confirmed low risks for the trisomies 13, 18 and 21, with a fetal fraction of 6%. The second trimester ultrasound (US) evaluation at 20 + 2 WG identified fetal growth restriction, an aberrant right subclavian artery (ARSA) and increased utero-placental resistance in the uterine arteries. An amniocentesis was proposed, and both the QF-PCR and the SNP-array on DNA extracted from the amniotic fluid cells were compatible with a T21 (Figures 1A,B). A second NIPT analysis was offered by the NIPT provider, which was performed at 20 + 3 WG, with a fetal fraction of 8%, and the results indicated a low risk for the main autosomal trisomies. The couple was given counselling by a multidisciplinary team for possible feto-placental mosaicism, after which they asked for a second amniocentesis, which confirmed the T21. The couple asked for termination of the pregnancy according to Italian legislation (Law 194/78).

**FIGURE 2:**
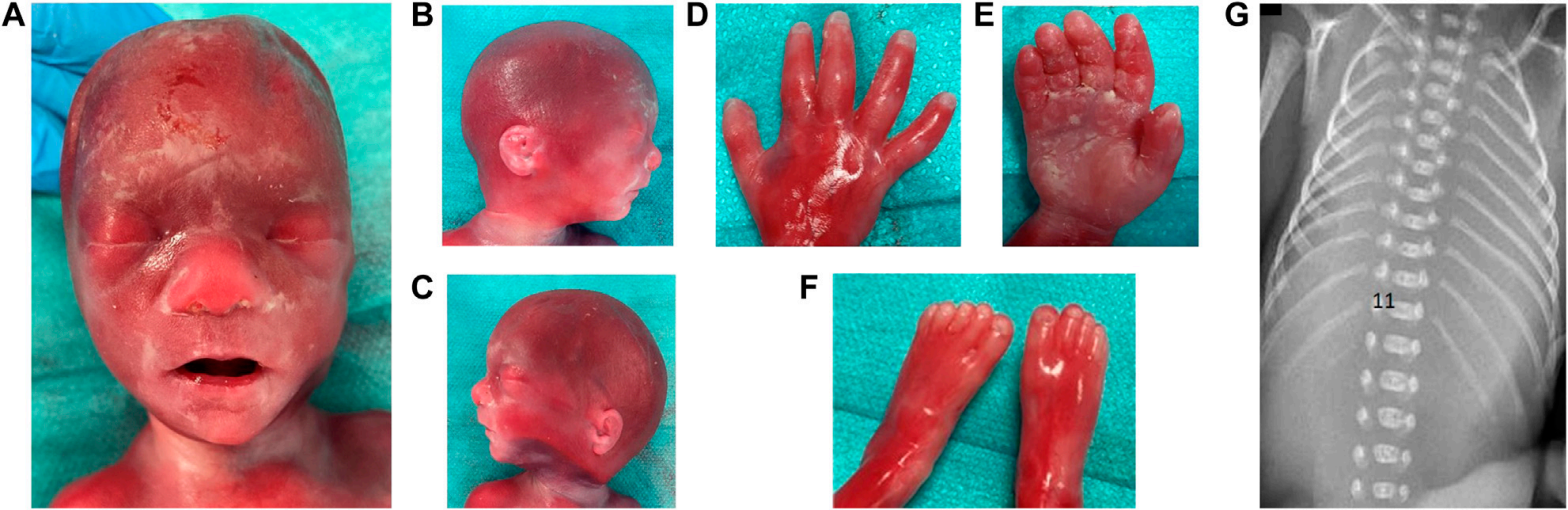
Dysmorphological examination of fetus of Case 1 is shown in **(A)** facial view: flat nasal bridge, prominent philtrum; **(B)** facial right; **(C)** facial left profile: ear asymmetry, mildly low-set ears, right lobar hypoplasia, mild retrognathia; **(D)** dorsal face of right hand: clinodactyly of 5th finger; **(E)** palmar face of right hand; **(F)** feet; **(G)** 11 bilateral and complete rib pairs and costal sketch of the twelfth vertebra on the right.

After expulsion at 21 + 5 WG, the fetal dysmorphological examination showed a flat nasal bridge, prominent philtrum, mild macroglossia, ear asymmetry, slightly low-set ears, mild retrognathia, bilateral clinodactyly of fifth fingers (Figures 2A–F). Post-mortem imaging also revealed the presence of 11 rib pairs (Figure 2G). The autopsy confirmed both the presence of ARSA and the radiologic findings, without other malformations. The placenta examination was unremarkable.

**FIGURE 3:**
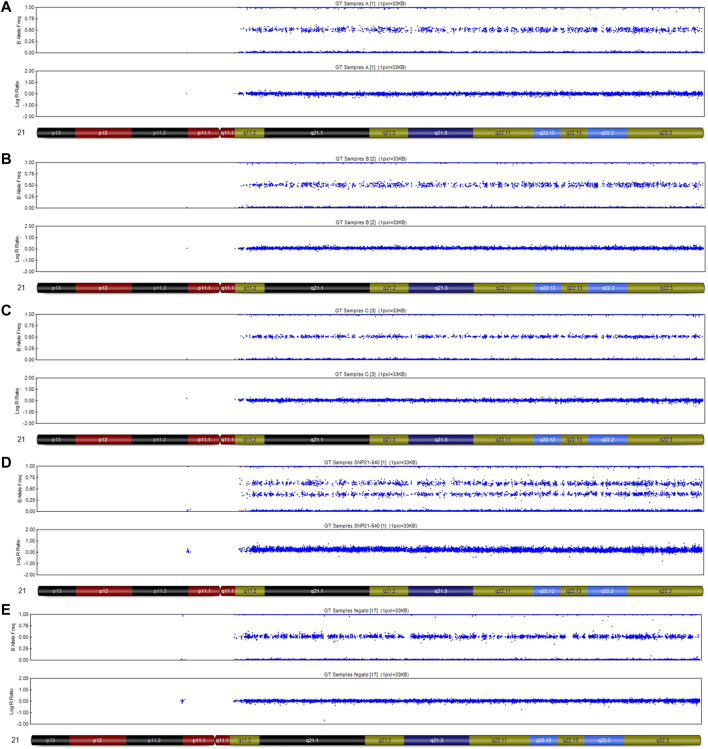
SNP-array analysis on placenta and fetal tissues of Case 1. **(A)** placenta sample 1 DNA, very low level of mosaicisms T21; **(B)** placenta sample 2 DNA, very low level of mosaicims T21; **(C)** placenta sample 3 DNA, disomy chromosome 21; **(D)** fetal skin DNA, complete T21; **(E)** fetal liver DNA, disomy chromosome 21.

Three placental samples were analysed via SNP-array, showing different results: two of the samples indicated the likelihood of a very low level of T21 mosaicism (Figures 3A,B), while a chromosome 21 disomy was evident in one sample (Figure 3C). DNA from fresh fetal skin showed a T21 (Figure 3D). DNA from the fetal liver showed the presence of euploidy (Figure 3E).

### Case 2

**FIGURE 4:**
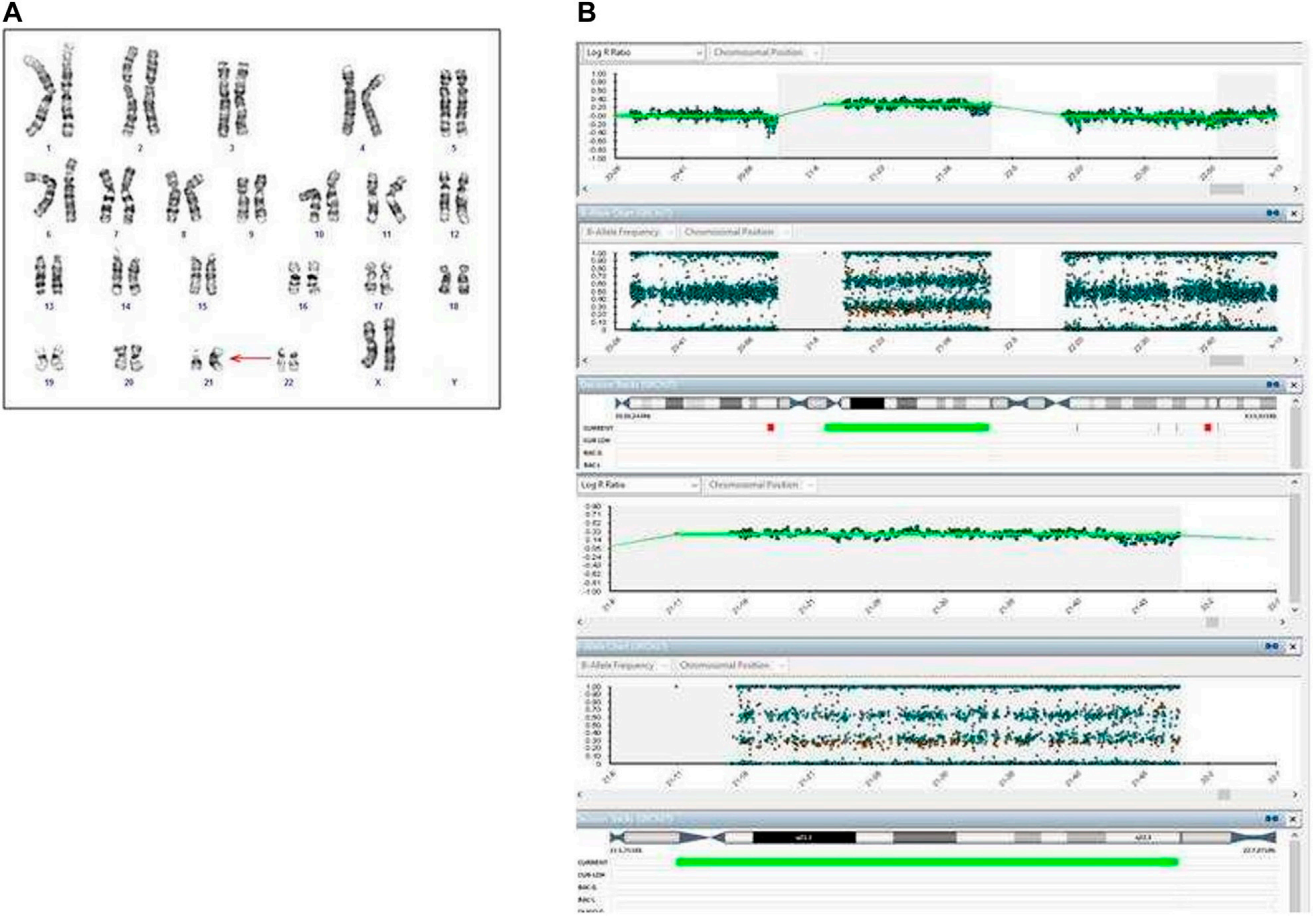
Cytogenetic and cytogenomic analyses on amniotic fluid of Case 2. **(A)** GTG banding karyotype shows a complete T21; **(B)** SNP-array shows a complete T21. Log ratio >0 indicates a copy number gain for chromosome 21.

The case involves the first spontaneous pregnancy of a healthy couple of Caucasian ancestry, with unremarkable personal and family history. The woman’s age at conception was 33. The first trimester echography performed a slightly increased NT (NT = 3.35 mm). The couple performed a NIPT at 10+5WG, which confirmed low risks for the trisomies 13, 18 and 21 and the absence of Y chromosome, with a fetal fraction of 8%. Increased NT value persisted at the following US evaluation and an amniocentesis was proposed and performed at 17 WG. Cytogenetic and SNP‐array analyses showed 46,XX,+21,der (21;21)(q10;q10) (Figure 4A) and a trisomy 21 (Figure 4B), respectively. A second cffDNA was performed at 18 WG and the results again showed the absence of common chromosomal trisomies. The couple was given genetic counselling and finally asked for termination of the pregnancy according to Italian legislation (Law 194/78).

**FIGURE 5:**
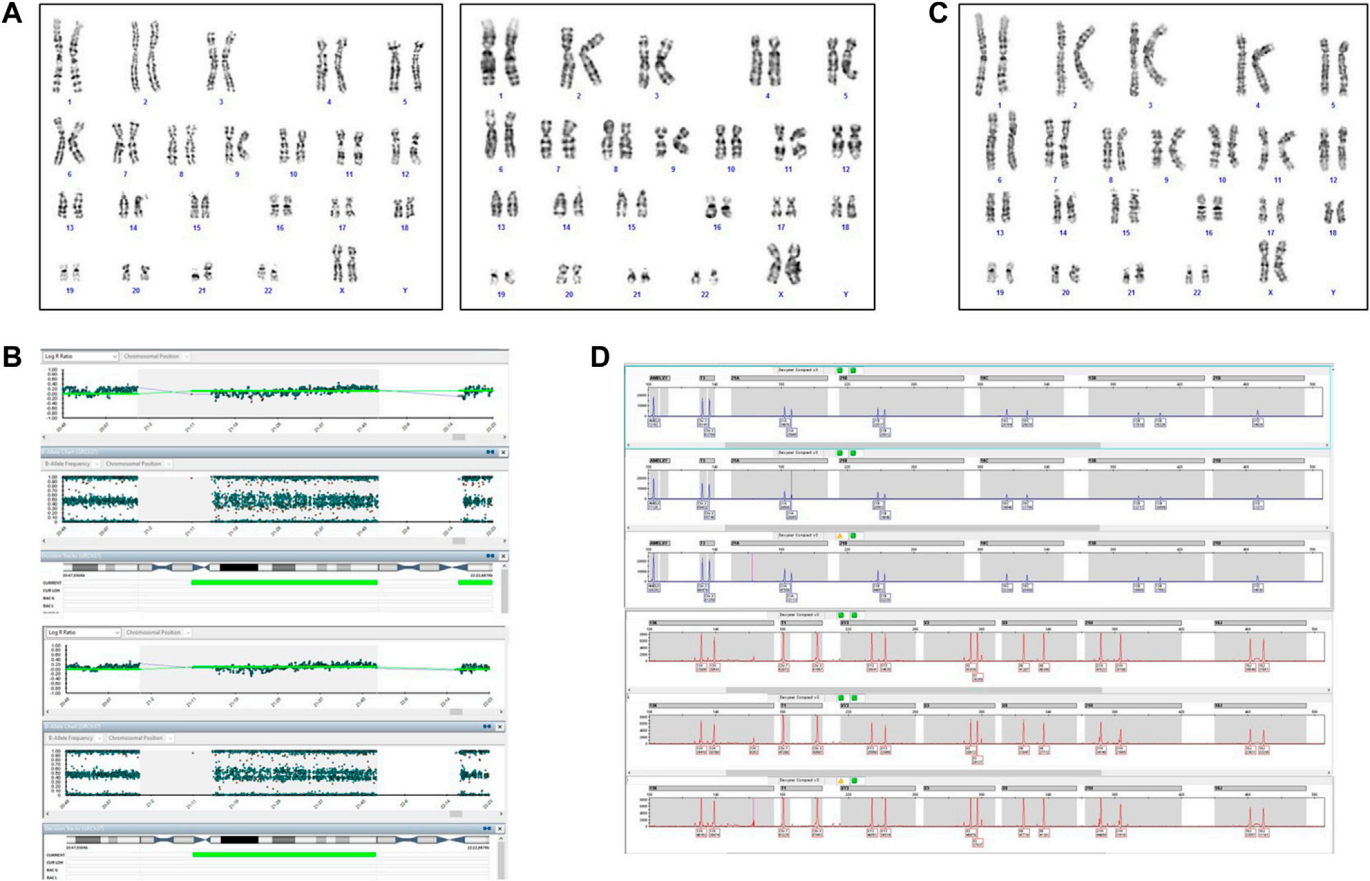
Cytogenetic and cytogenomic analyses on placenta and fetal tissues of Case 2. **(A)** GTG banding karyotype on placenta shows a mosaic T21; **(B)** SNP-array on two samples of placenta shows a mosaic T21. Log ratio >0 indicate a copy number gain for chromosome 21; **(C)** GTG banding karyotype on fetal skin shows a complete T21; **(D)** QF-PCR analysis on placenta. For each channel, the three lanes represent the different biopsies from the placenta.

After expulsion at 20 + 5 WG, the fetal dysmorphological examination showed an eutrophic female fetus with macroglossia, low set ears, hypertelorism and micrognathia. The autopsy confirmed normal intrathoracic, intraabdominal and pelvic organs. The placenta showed no macroscopic or histologic anomalies. The placenta and fetal tissues were further examined via cytogenetic and cytogenomic analyses. Karyotype and SNP‐array analyses revealed a placental mosaicism at about 60% (Figure 5A,B), while the SNP‐array on DNA fetal skin showed a complete T21 (Figure 5C).

The QF-PCR patterns on placental tissue were consistent with the fetus’ disomy for 13, 18, trisomy for 21, and the presence of two X chromosome and absence of the SRY gene (Figure 5D). In particular, all information carrying autosomal short tandem repeats markers demonstrated a normal 1:1 marker ratio, while sexual chromosome markers resulted compatible with a female genotype. The chromosomes markers for T21 (21B and 21H for example, as shown in Figure 5D) denote the presence of two cell lines, i.e., one disomic and one trisomic cell line for T21. An investigation of the two cell lines showed that placental mosaicism was present at about 50%.

## Materials and methods

Case 1 and case 2 were referred to the Unit of Fetal Medicine and Prenatal Diagnosis and the Medical Genetics Laboratory at the Institute for Maternal and Child Health, IRCCS “Burlo Garofolo” in Trieste and to AMES laboratory in Naples and Medical Genetics in Avellino for prenatal consultation and analysis, respectively. Fetal autopsy was performed at the Pathological Anatomy and Histology Department of ASUGI (Trieste, Italy) and at Hospital of Avellino, for case 1 and case 2, respectively. Written informed consent for genetic analysis, clinical research and scientific publication were obtained according to the ethical standard defined by the Helsinki declaration.

### NIPT analysis

For NIPT analysis, about 10 ml of peripheral blood was collected from the pregnant women in Streck blood collection tubes. For plasma isolation, the blood sample was first centrifuged at 1,600 g for 10 min at 4°C to separate the plasma from peripheral blood cells. Cell-free DNA from 900 μL of maternal plasma was extracted using the QIAamp DNA Blood MiniKit (Qiagen, Hilden, Germany) following the manucfacturer’s protocol. NIPT analysis was performed using the VeriSeq NIPT Solution v2 bioinformatic pipeline (Illumina Inc., San Diego, CA, United States) based on the paired-end sequencing technique. The assay can report the results as Basic, with reporting for common trisomies and sex chromosomes (if selected), and Genome-wide analysis if the detection of the genome-wide fetal anomalies were included (including rare autosomal aneuploidies and partial deletions and duplications ≥ 7 Mb) (Borth et al., 2021; Pertile et al., 2021). The VeriSeq NIPT Assay Software v2 (www.illumina.com/NIPTsoftware) was used for data analysis of the aneuploidy status and fetal fraction from cffDNA. Sample results were classified using the VeriSeq NIPT Solution v2 Assay Software and analysis of “raw data” as reported previously (Borth et al., 2021; La Verde et al., 2021).

### DNA extraction

Amniotic fluid, women’s blood samples, placenta and fetal tissues were collected. Genomic DNA was extracted using the EZ1 (QIAGEN Hilden, Germany) automated system. The extraction process was performed according to the instructions on the kit. After extraction, the quality and quantity of DNA were analysed using QIAxpert spectrophotometry.

### QF-PCR

In the case 1 the test was performed using the Multiplex PCR Devyser kit (Devyser, Stockholm, Sweden), which was tested on 26 markers, including five STRs from chromosome 13 (D13S742, D13S634, D13S628, D13S305, D13S1492), five from chromosome 18 (D18S978, D18S535, D18S386, D18S976, GATA178F11), six from chromosome 21 (D21S1435, D21S11, D21S1411, D21S1444, D21S1442, D21S1437), and ten STRs from chromosomes X and Y (DXS1187, XHPRT, DXS2390, SRY, DXYS267, DXYS218, AMELX, AMELY, ZFY, ZFX). All the markers and the labelling information are included in the Devyser user manual. The PCR reaction was carried out using the SimpliAmp Thermal Cycler (Thermo Fisher, Waltham, MA, United States). Fragment analysis was performed through capillary electrophoresis using the Applied Biosystems 3,500 Dx DNA sequencer (Thermo Fisher, Waltham, MA, United States) after calibration, according to the instructions on the kit. The samples were run on a POP7 polymer. The results were analysed using GeneMapper™ Software (Thermo Fisher, Waltham, MA, United States). In the case 2 the quantitative fluorescent polymerase chain reaction test for rapid aneuploidy detection was performed using the Devyser Compactv3 QF-PCR kit (QF-PCR; Devyser Compactv3, Devyser). The amplified DNA samples were separated through electrophoresis using the ABI 3130xl Genetic Analyzer, and each allele was analysed for specific markers using GeneMapper Software ver. 4.0 (Applied Biosystems).

### Cytogenetic analysis

For case 2, chromosomal analysis was performed on long-term amniotic fluid cultures from two separate culture flasks. GTG-banding 40 metaphases were analysed using CytoVision software (CytoVision, AB Imaging).

### SNP‐array

In the case 1 the SNP-array analysis was performed on the genomic DNA using the Human OmniExpress Exome-8 Bead Chip (Illumina Inc., San Diego, CA, United States), which contains 960,919 loci derived from phases I, II and III of the International HapMap project. The array contains over 274,000 functional exonic markers, delivering unparalleled coverage of putative functional exonic variants selected from 12,000 individual exome and whole-genome sequences. In the case 2 the SNP‐array analysis was performed on the genomic DNA using the HumanCytoSNP-12 v12.1 BeadChip Kit (Illumina Inc., San Diego, CA, United States), which contains ∼300,000 SNPs targeting regions.

A total of 200 ng of gDNA (50 ng/μl) for each sample was processed according to Illumina’s Infinium HD Assay Super protocol. The normalization of raw image intensity data, genotype clustering and individual sample genotype calls were performed using Illumina’s GenomeStudio software v2.0 (cnvPartition 3.2.1). The CNVs were mapped to the human reference genome hg19 and UCSC refGene was used to annotate the gene variation. Allele detection and genotype calling were performed using GenomeStudio and NxClinical software.

### Formalin-fixed paraffin-embeded (FFPE) samples

During fetal autopsy, several organ samples were collected and fixed in formalin. The only tissue eligible for analysis in case 1 was a liver biopsy, which was retrieved from the paraffine sample using a scalpel and DNA was then extracted using the QIAamp® DNA FFPE kit (QIAGEN, Hilden, Germany). After extraction, the quality and quantity of DNA were analysed using Quiaxpert spectrophotometry coupled with the Qubit dsDNA BR Assay Kit (Invitrogen) fluorimetry.

Prior to the SNP-array analysis, the sample was restored with the Infinium HD FFPE DNA Restore Kit (Illumina, San Diego, CA, United States) and the quality was newly assessed using the Qubit dsDNA BR Assay Kit. This kit enhances the quality of FFPE-extracted DNA, which is known to be fragmented, and enables an optimized whole-genome amplification strategy.

## Discussion

### Feto-placental mosaicism

Mosaicism is a biological condition in which two or more cell lines with different karyotypes derived from a single zygote coexist in a single individual (Strachan and Read, 2019). Most frequently, one of the cell lines may present a complete or partial aneuploidy and/or a chromosomal structural rearrangement (Porter et al., 1999; Brisset et al., 2003; Soler et al., 2003). Chromosomal mosaicism is one of the primary interpretative issues in prenatal diagnosis and it is diagnosed through villocentesis and amniocentesis, in 1–2% and 0.1–0.3% of pregnancies, respectively (Ledbetter et al., 1992; Smidt-Jensen et al., 1993; Malvestiti et al., 2015). Apart from the type of chromosome mechanism, the distribution of the different cell lines in the fetus and/or the placenta depends also on the timing when the mosaicism occurred and, on the embryo/fetal localization. Mosaicism could involve 1) only the placenta, where the condition is known as “confined placental mosaicism”; 2) both the placental and the fetus, where the condition is “feto-placental mosaicism” (Grati, 2014; Grati et al., 2017); 3) the fetus only. Thus, a complete fetal T21 may coexist with a normal placenta, a placenta with a complete trisomy or a placenta with a placental mosaicism (Eggenhuizen et al., 2021). These conditions may result in different clinical manifestations and diseases (Thorpe et al., 2020).

In that it is a screening test, the NIPT result requires confirmation through an invasive analysis (Hartwig et al., 2017). Chorionic villus sampling, or villocentesis, examines the cytotrophoblast and syncytiotrophoblast cells of the placenta, while amniocentesis analyses the fetal amniotic fluid cells, representing fetal tissues. Amniocentesis is the gold standard to confirm or exclude the NIPT result (Grati, 2014) in case of a high risk of T21. NIPT and the invasive test can potentially give discordant results, for various reasons, including false negative NIPT results as a consequence of mosaicism. The presence of feto-placental mosaicism can affect the interpretation and management of NIPT results, an issue given that the test is generally taken to determine the risk of fetal chromosome aneuploidies. Indeed, feto-placental mosaicism can generate discordance between the results from cffDNA testing and amniocentesis, producing a “false negative” or “false positive” NIPT result (Grati, 2014). The NIPT detection rate for T21 typically exceeds 99% with a low false-positive (FP) rate (<0.1%) (Mackie et al., 2017) and rare false-negative (FN) cases reported in some clinical studies (Zhang et al., 2015). To date, various FN T21 cases have been reported (Huijsdens-van Amsterdam et al., 2018), indicating that FN NIPT results may occur through biological mechanisms rather than through technical limitations.

Therefore, distinguishing between these embryologically and biologically different situations is challenging, and requires specific sampling and cytogenetic and cytogenomic analyses in various tissues. With the wide-spread implementation of prenatal non-invasive and invasive testing and the emergence of discrepant results, it may be helpful to set up strategies for investigating and understanding the mosaicism mechanism, and so back the counselling of couples and management of pregnancies with the right information. Genetic and chromosomal conditions of the placenta may differ from those of the fetus for different reasons (Hartwig et al., 2017). Thus, a negative NIPT result can only exclude the majority of adverse copy number changes in the fetus and/or the placenta, and a positive NIPT for a trisomy can be a false positive in up to ∼2% of cases. Confined placental mosaicism should be presented as a real and serious condition to couples, who should be properly informed about the interpreting of NIPT and screening findings preferably before taking the tests, and they should be offered pre- or post-test counselling (Lau et al., 2014; Hartwig et al., 2017; Liehr et al., 2017). More recent developments in studying fetal and placental cell trafficking into the maternal circulation includes fetal cell based NIPT (cbNIPT), consisting in the examination of specifically extravillous trophoblasts originating from the placenta. This strategy is actually experimentally performed, having different limitations mainly due to accessibility and costs (Vossaert et al., 2021). Despite further studies are needed to assess its validity, it has proved, for example, a potential role in the screening of maternal mosaicism of sex-chromosomes anomalies, being superior to cell-free NIPT, which could fail to discriminate between maternal or fetal mosaicism (Jeppesen et al., 2021). In the context of false negative results at cell-free NIPT, analysing the total amount DNA, the results from cbNIPT, analysing only single cells or pool of cells harvested from maternal blood miming a placenta biopsy, could be candidate in the detection of confined placental mosaicism.

We have presented here two cases of feto-placental mosaicism, which were postulated after a discordant result between cell-free NIPT and amniocentesis and confirmed through cytogenetic and cytogenomic analyses of placental and fetal tissues. One of the main challenges in investigating mosaicism is to establish at which point during the embryo-fetal development does the mitotic error occur (Grati et al., 2017).

### The issue of diagnosis

In the first case, the clinical presentation was not specific of aneuploidy, while in the second case, the increased NT was an early sign of suspect. However, in both cases, the diagnosis of T21 was clearly confirmed in the second trimester. In the second case, although the increased NT found in the first trimester of pregnancy suggested that an invasive test was advisable, the pregnant woman preferred to undergo a NIPT test, which recorded low-risk results for common trisomies. The NT measurement in combination with serum biomarkers and maternal age meant that she was offered FTCS programme (Snijders et al., 1998). The introduction of non-invasive prenatal testing (NIPT) makes it possible to obtain information for common trisomies from as early as 10 weeks into gestation, proposing an alternative to traditional FTCS (Hui and Bianchi, 2017). Despite the superiority of NIPT to the combined test in the detection of T21, the fetal sonographic assessment is crucial for the determination of NT (Bardi et al., 2020). In the second case, the persistence of an increased NT measurement with a low-risk NIPT result was a challenge for both clinicians and the couple. In this context, an enlarged NT could be a marker for genetic conditions and fetal anomalies that would not be detected through NIPT (Yagel, 2021), as well as a condition of a feto-placental mosaicism. In our case the findings of the ultrasound subsequent to low risk cffDNA screening resulted in the woman deciding to undergo amniocentesis, and a non-mosaic isochromosome 21 was found (Oepkes et al., 2016).

Various elements characterizing the first case, such as the low PAPP-A value with a low-risk FTCS, mild ultrasound signs and ARSA, together with a few syndromic dysmorphisms and skeletal features with no major malformations, are valuable discussion points and clues for the diagnosis of T21 mosaicism.

A reduced PAPP-A level, which is the primary biochemical marker for the most common trisomies, in particular for T21 (Fialova and Malbohan, 2002), was the only altered marker at FTCS, when no suspicion of a possible fetal concern had emerged. Retrospectively, this serum marker may have been evocative of a placental mosaicism, and have contributed both to fetal growth restriction and reduced placental weight (Yong et al., 2009; Eggenhuizen et al., 2021).

Arguments supporting a possible fetus concern emerged during the second trimester US evaluation, highlighting fetal growth restriction, increased uterine arteries resistance and ARSA. In this case, maternal smoking could have primarily contributed to reducing the PAPP-A value, considering the periconceptional and pregnancy exposition to tobacco (Spencer, 1999), as well as to increasing arterial uterine resistance (Pintican et al., 2019) and directly and consequently to determining a fetal growth restriction (Abraham et al., 2017). Increased uterine resistance is more common in pregnancies with T21 than in normal (euploidy) pregnancies (Kaur et al., 2021). Fetal growth restriction is a common complication of pregnancies and could be a sign of a number of pathological conditions, including T21 (Fetal Growth Restriction: ACOG Practice Bulletin, Number 227, 2021). ARSA is the most common abnormality of the aortic arch, both in the general population and in normal (euploid) fetuses, affecting respectively 1–1.5% and 0.4–1.5%, with about a 16% prevalence in fetuses with T21 (Scala et al., 2015; Martínez-Payo et al., 2022). Isolated markers during the second trimester US evaluation, such as isolated ARSA, have a small effect on modifying the screening pre-test odds for T21, but all the most recent evidence suggests that ARSA should be considered as a soft marker of T21 (Agathokleous et al., 2013; Martínez-Payo et al., 2022).

Despite the low FTCS risk, with the nasal bone visualization and a negative NIPT result, the additional association of minor signs, as likely soft markers, which emerged in the second trimester, led to the multidisciplinary consultation and the indication for performing an amniocentesis, which demonstrated the presence of T21.

### The issue of post-mortem analysis

In both cases, post-mortem examinations brought up other elements, including phenotypic features (Radhakrishnan et al., 2018), typical craniofacial anomalies (Guihard-Costa et al., 2006) and minor skeletal findings consistent with the diagnosis of T21. The dysmorphological examination revealed and supported the diagnosis of T21 in the fetus, while the total body imaging showed the presence of 11 pairs of complete ribs, which is a skeletal finding in 11% of fetuses with T21 investigated by autopsy (Grangé et al., 2006) and 33% of radiologically investigated newborn infants with T21 (Edwards et al., 1988). When there is a T21 diagnosis, there will also be a discussion as to whether a fetal autopsy should or should not be performed, in that it is complementary to the prenatal US investigation (Papp et al., 2007). Additionally, when there is such a T21 diagnosis, post-mortem cytogenomic analyses are uncommon, and DNA tests on fetal or placental tissues are, in general, never carried out. However, the indication for an autopsy was discussed with both the reported couples, and they gave their consent for classical and molecular autopsy (DNA analyses from fetal tissues). Cytogenetic and/or cytogenomic analysis were performed in placenta and fetal tissues in both cases, providing the confirmation of mosaicism in both the placenta and the fetus in the first case, and only in the placenta with complete T21 in the fetus in the second case. It is likely that the generation of the mosaicism was an early event in both cases. Indeed, in a feto-placental mosaicism, the postzygotic mitotic error, being itself a nondisjunction event in a somatic cell of an euploid conceptus or a trisomy rescue after a meiotic nondisjunction, will probably occur very early after the zygote has formed, that is, before the separation between embryonic and extraembryonic tissues (Grati, 2014). It has been shown that among the FN cases of T21 at NIPT, previously mentioned, about 22% had an isochromosome 21q, 44% had T21 and only one case had a feto-placental mosaicism (Huijsdens-van Amsterdam et al., 2018). As the cytotrophoblast is the primary source of the “fetal” cffDNA, a *de novo* isochromosome 21q formation which occurs in the inner cell mass precursors will be seen in the mesenchymal core and fetus, but, since the cytotrophoblast cells would remain predominantly euploid, the chromosomal aberration could not be detected via NIPT (Flori et al., 2004). A further difficulty is due in the absence, in many cases, of any studies on placenta and/or fetal tissue. A post-mortem molecular autopsy has thus revealed to be an effective strategy to explain the molecular identity of placental and fetal tissues allowing to better define the typology of mosaicims. Finally, it could be an effective method to discriminate between placental, fetal or feto-placental mosaicism, and between complete or mosaic fetal chromosomal anomalies in cryptic prenatally uninvestigated conditions, as well as in antenatally undiagnosed cases.

### The issue of counselling to couples

Both the cases reported here emphasize that there is the need for accurate and complete pre-test NIPT counselling. Couples should be informed about the meaning of NIPT as an accurate screening test, about its technical and biological limitations and about the possibility of false positive and false negative results (Liehr, 2021). Positive NIPT results always lead to a consultation being offered and require prompt investigations for their confirmation (“true positive”) or confutation (“false positive”). On the contrary, a false negative NIPT result may be more difficult to find and investigate, or worse, it may falsely reassure both clinicians and couples, who may underestimate the risk of potential pathological conditions, such as feto-placental mosaicisms or fetal trisomy.

A multidisciplinary approach in counselling, as well as in the interpretation of biological events, is essential to explain complex cases, such as feto-placental mosaicisms.

In case of feto-placental mosaicisms, the lethality of a chromosomal aneuploidy is expected to be attenuated and related to the amount of trisomic cells and their distribution in different organs. In the prenatal setting, the mosaicism is quantified basing on fetal cells in the liquid amniotic, and thus of a partial proportion of fetal systems. A precise prediction about the postnatal clinical presentation is not possible. In particular, the severity of intellectual disability and eventual associated neuropsychiatric concern, as well as the prediction of sensorial deficits, which are typical of T21 is never feasible. US examination gives accurate description of structural anomalies, contributing to orient the prognostic outcome in terms of possible life-threating concerns at birth and indication of dedicated setting for delivery. The significance of mosaicism as a “Variant of Uncertain Outcome” (Levy et al., 2021) is one of the main challenging messages to provide during counselling of couples in the prenatal setting.

## Data Availability

The datasets for this article are not publicly available due to concerns regarding participant/patient anonymity. Requests to access the datasets should be directed to the corresponding author.
